# Expanding the Scope of Non-invasive Prenatal Testing to Detect Fetal Chromosomal Copy Number Variations

**DOI:** 10.3389/fmolb.2021.649169

**Published:** 2021-05-12

**Authors:** Songchang Chen, Lanlan Zhang, Jiong Gao, Shuyuan Li, Chunxin Chang, Yiyao Chen, Hongjun Fei, Junyu Zhang, Yanlin Wang, Hefeng Huang, Chenming Xu, Daru Lu

**Affiliations:** ^1^State Key Laboratory of Genetic Engineering, MOE Engineering Research Center of Gene Technology, School of Life Sciences, Fudan University, Shanghai, China; ^2^Genetics Center of Obstetrics and Gynecology, Obstetrics and Gynecology Hospital, Fudan University, Shanghai, China; ^3^The International Peace Maternity and Child Health Hospital, School of Medicine, Shanghai Jiao Tong University, Shanghai, China; ^4^Shanghai Key Laboratory of Embryo Original Diseases, Shanghai, China; ^5^Shanghai Municipal Key Clinical Specialty, Shanghai, China; ^6^Institutes of Biomedical Sciences, Fudan University, Shanghai, China; ^7^Shanghai Medical Laboratory, BGI-Shanghai, BGI-Shenzhen, Shanghai, China; ^8^Key Laboratory of Birth Defects and Reproductive Health of National Health Commission, Chongqing Population and Family Planning, Science and Technology Research Institute, Chongqing, China

**Keywords:** non-invasive prenatal testing, copy number variation, sequencing depth, microdeletion/microduplication syndromes, next generation sequencing

## Abstract

Non-invasive prenatal testing (NIPT) for common fetal trisomies is effective. However, the usefulness of cell-free DNA testing to detect other chromosomal abnormalities is poorly understood. We analyzed the positive rate at different read depths in next-generation sequencing (NGS) and identified a strategy for fetal copy number variant (CNV) detection in NIPT. Pregnant women who underwent NIPT by NGS at read depths of 4–6 M and fetuses with suspected CNVs were analyzed by amniocentesis and chromosomal microarray analysis (CMA). These fetus samples were re-sequenced at a read depth of 25 M and the positive detection rate was determined. With the increase in read depth, the positive CNV detection rate increased. The positive CNV detection rates at 25 M with small fragments were higher by NGS than by karyotype analysis. Increasing read depth in NGS improves the positive CNV detection rate while lowering the false positive detection rate. NIPT by NGS may be an accurate method of fetal chromosome analysis and reduce the rate of birth defects.

## Introduction

Since the first detection of cell-free fetal DNA (cffDNA) in the plasma of pregnant women in 1997 (Lo et al., 1997), comparisons of the chromosome distribution of cffDNA between patients and controls have played an increasingly important role in fetal aneuploidy diagnosis (Wang et al., 2006). Non-invasive prenatal testing (NIPT) as an alternative screening method has been shown to have very high sensitivity and specificity for detecting common chromosomal aneuploidies such as trisomy 13 (T13), trisomy 18 (T18), and trisomy 21 (T21) with low false positive and false negative rates (Song et al., 2013; Bianchi et al., 2014; Chandrasekharan et al., 2014; Liao et al., 2014). NIPT calculates the risk of fetal chromosomal aneuploidies by detecting cffDNA circulating in maternal plasma using next-generation sequencing (NGS) technology (Chandrasekharan et al., 2014).

Microdeletion/microduplication syndromes (MMS), which are due to copy number variations (CNVs), are another major inheritance factor that causes birth defects in newborns. CNVs are stretches of genomic DNA present as more than or fewer than two copies and can range in size from kilobases to megabases (Mb). They cannot be identified by G-banded chromosome analysis (karyotyping) but can be identified by microarray methodologies and whole genome sequencing analysis (Wang et al., 2017). CNVs are widespread in the human genome. The incidence of pathogenic CNVs in the normal population can reach 1.0–1.7%, which is much higher than the incidence of T21 (0.13–0.17%) (Wapner et al., 2012). More than 1,400 CNV regions have been found in European, African, and Asian populations. Approximately 14.50% of genes associated with known human genetic diseases exist in these CNVs (Redon et al., 2006). Because of the high incidence of fetal pathogenic CNVs and their severe clinical symptoms, feasibility studies of CNV detection in NIPT, which have been repeatedly proposed by the American College of Medical Genetics and American College of Obstetricians and Gynecologists, are becoming increasingly urgent.

Most existing studies on fetal CNV detection have focused on small samples. Peters et al. (2011) detected by NIPT a 4.2-Mb microdeletion on chromosome 12 in one fetus and validated it by extracting amniotic fluid and using array-based comparative genomic hybridization (aCGH). In 2012, Jensen et al. retrospectively validated by NIPT two 22q11.2 microdeletion samples with high cffDNA concentrations (17%–18%)(Oskarsdottir et al., 2004). Jia et al. (2014) performed routine NIPT on a pregnant woman at 17 weeks and found a suspected 3.71-Mb microdeletion in the fetus. The aCGH method was used to verify that the microdeletion was inherited from the mother. The detection of CNVs requires a higher cffDNA concentration and greater read depth than that of common chromosomal aneuploidies. In conjunction with the progress in cffDNA extraction technology and reduction in sequencing cost, it is possible and necessary to detect chromosomal microdeletions, microduplications, and pathogenic CNVs by NIPT on a large scale.

Chromosomal microarray analysis (CMA) has become the first-tier technique for the genetic follow-up of fetal structural anomalies identified by ultrasonography (South et al., 2013; Grati et al., 2015; Zhu et al., 2016). More recently, NGS has emerged as a high-resolution technology for genome-wide CNV detection (Liang et al., 2014; Cohen et al., 2015; Justino et al., 2015; Liu et al., 2015) and may represent another comprehensive approach for detecting pathogenic CNVs. However, the difference between CMA and NGS in CNV detection is not clear, which causes issues for clinicians.

The aim of this study was to analyze the positive CNV detection rate at different read depths (4–6 and 25 M) of cffDNA using NGS and compare the positive detection rates with those of amniocentesis and CMA to determine the most efficient method of identifying CNVs. The findings of this study could help improve diagnostic accuracy while reducing cost and improving maternal and prenatal health care.

## Results

### Demographic Details of Enrolled Patients

**FIGURE 1:**
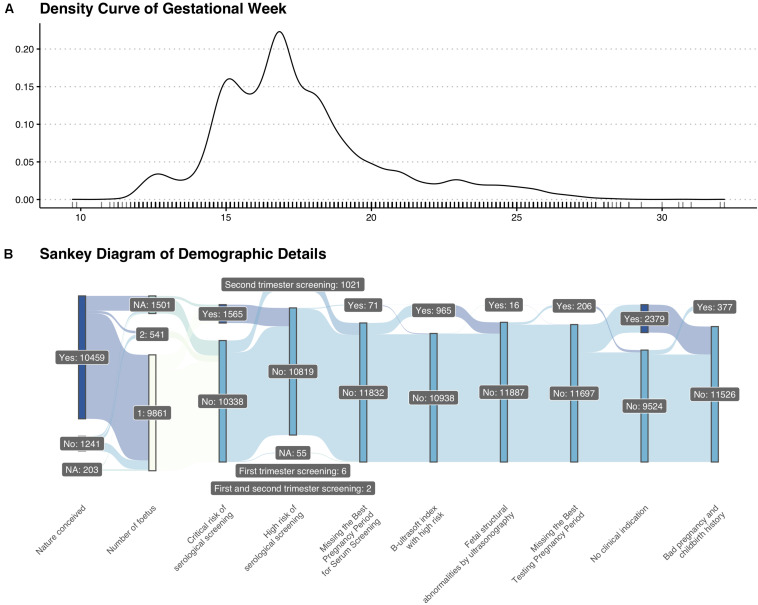
Basic information of enrolled patients. **(A)** Density curve of gestational weeks. **(B)** Sankey diagram of demographic details.

A total of 11,903 pregnant women were enrolled in our research. The average gestational week was around 17 + 3 weeks. The density curve of gestational week and detailed demographic features were shown in Figure 1.

### Diagnoses of MMS by NIPT on BGISEQ-500 at a Read Depth of 6 M

**FIGURE 2:**
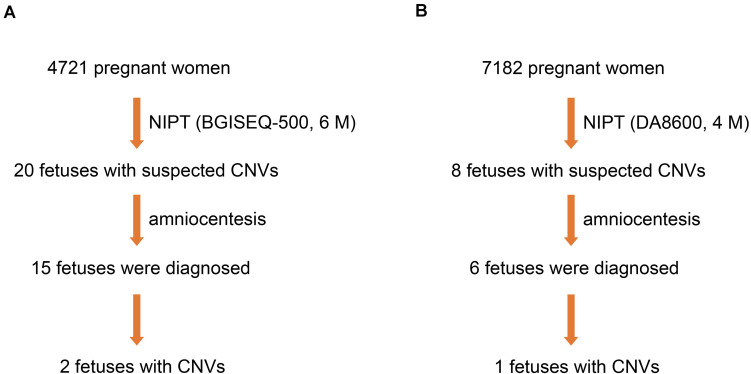
Three fetuses diagnosed with MMS. **(A)** Two fetuses were diagnosed by NIPT based on the BGISEQ-500 sequencing platform with a read depth of 6 M and subsequent amniocentesis. **(B)** One fetus was diagnosed by NIPT based on the DA8600 sequencing platform with a read depth of 4 M and subsequent amniocentesis.

From March 2017 to June 2018, 4,721 pregnant women underwent NIPT by NGS on the BGISEQ-500 sequencing platform at a read depth of 6 M. We found 20 fetuses (0.42%) with suspected CNVs. Subsequently, 15 fetuses (75.0%) were further evaluated by amniocentesis and CMA, two of which (13.3%) were diagnosed with MMS (Figure 2A).

### Diagnosis of MMS by NIPT on DA8600 at a Read Depth of 4 M

To compare the efficiency of different sequencing platforms in CNV detection, we analyzed 7,182 pregnant women who received NIPT by NGS on the DA8600 sequencing platform at a read depth of 4 M. Suspected CNVs were detected in eight fetuses (0.11%), and six of the eight fetuses (75.0%) were further evaluated by amniocentesis and CMA. However, only one of the six fetuses (16.7%) was diagnosed with MMS (Figure 2B). In the process of fetal CNV detection, low read depth (4–6 M) led to a low positive rate (3/21, 14.3%) and a high false positive rate (18/21, 85.7%).

### NIPT Based on BGISEQ-500 at a Read Depth of 25 M

**FIGURE 3:**
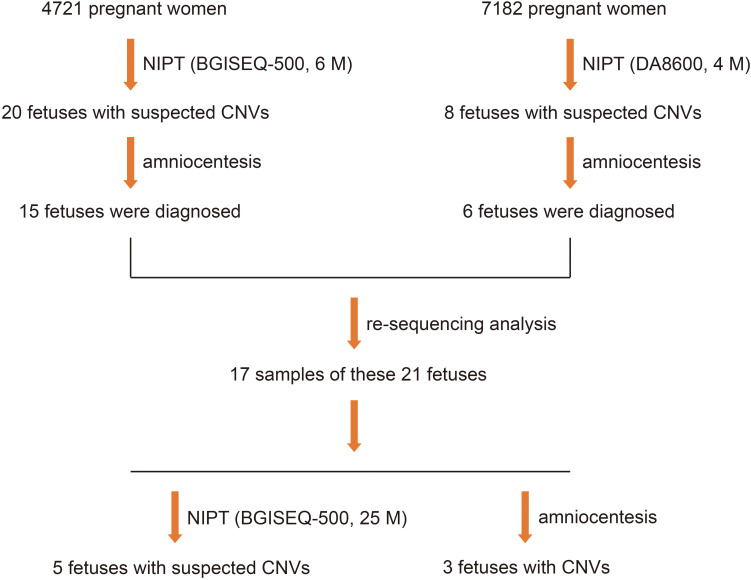
Five positive results in 21 fetuses with suspected CNVs, detected using NGS at read depths of 4–6 M first and then by NGS on the BGISEQ-500 sequencing platform at a read depth of 25 M.

To clarify whether increasing the depth of sequencing helps to increase the positive rate and reduce the false positive rate of fetal CNV detection, we sequenced the samples of 21 fetuses with suspected CNVs at a read depth of 25 M on the BGISEQ-500 sequencing platform and found that 17 met the requirements for re-sequencing. Five positive results of suspected CNVs were detected, which showed that, with the increase in read depth from 4–6 to 25 M, the positive rate of fetal CNV detection increased from 14.3 to 60.0% (Figure 3).

### Role of Read Depth in Fetal CNV Detection

**FIGURE 4:**
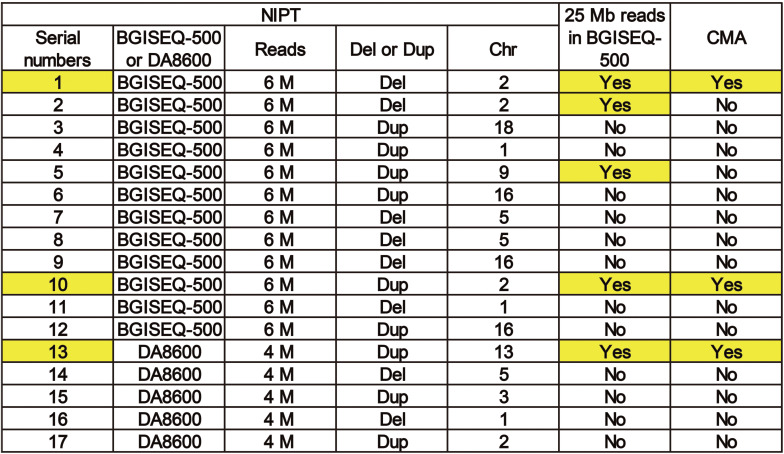
A list of 17 cases that met the requirements for re-sequencing at 25 M reads on theBGISEQ-500 sequencing platform.

**FIGURE 5:**
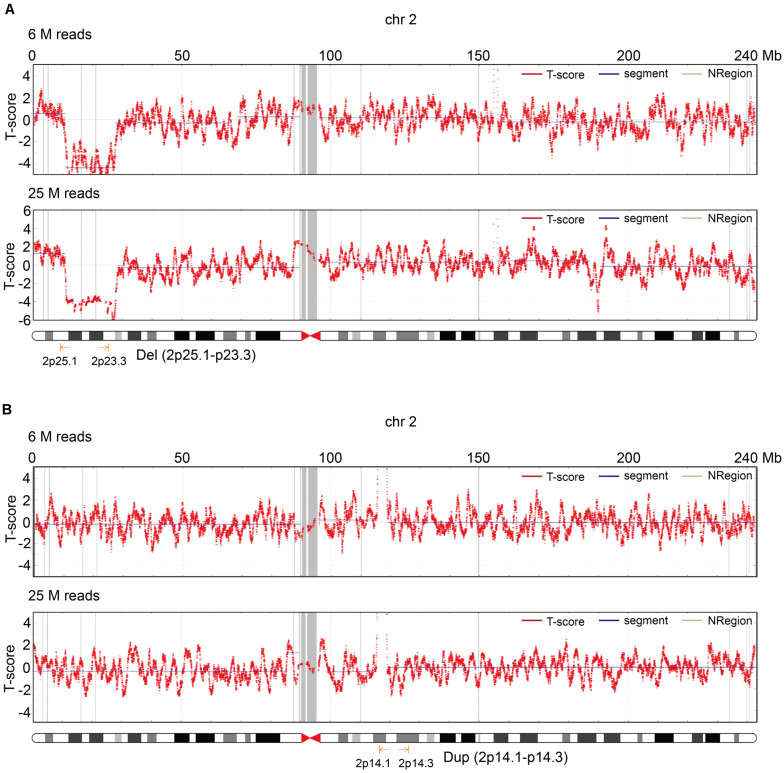
Two cases that illustrate the role of read depth for fetal CNV detection. **(A)** *T*-score of chr2 in the read depth of 6 and 25 M in the first case. **(B)** *T*-score of chr2 in the read depth of 6 and 25 M in the second case.

To improve the understanding of how read depth improves the positive fetal CNV detection rate, we listed 17 cases that met the requirements for re-sequencing with 25 M reads on the BGISEQ-500 sequencing platform (Figure 4) and took two specific cases for detailed analysis (Figure 4). The read depth of 25 M had a higher *T*-score and greater detection of CNVs [del (2p25.1–p23.3) or dup (2p14.1–p14.3)] than the read depth of 6 M (Figure 5). Importantly, no obvious signs of different abnormalities were observed in other chromosomes between the read depths in these positive cases.

### Positive CNV Detection Rates in NGS With 25 M Reads and in CMA

To compare the diagnostic concordance of CMA and NGS, we sequenced 88 fetus samples with CNVs identified by CMA at 25 M reads in NGS and analyzed the rate of positive CNV detection in CMA and NGS. Because some fetuses carried two or more types of CNVs, we actually checked 102 CNVs in these 88 cases.

**FIGURE 6:**
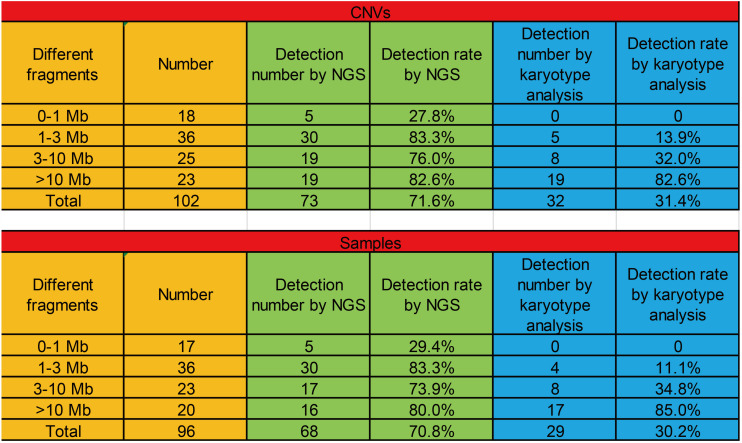
The positive detection rates of CNVs with different fragments (0–1, 1–3, 3–10, and >10 Mb) among CMA, NGS, and karyotype analysis.

Because these cases involved different diseases, they were generally categorized into four groups by different fragment sizes (0–1, 1–3, 3–10, and >10 Mb). The positive CNV detection rates with 0–1, 1–3, 3–10, and >10 Mb fragments were 27.8, 83.3, 76.0, and 82.6%, respectively, and the positive detection rates in samples with 0–1, 1–3, 3–10, and >10 Mb fragments were 29.4, 83.3, 73.9, and 80.0%, respectively. Then, we checked the detection rates in these cases by karyotype analysis. The positive CNV detection rates with 0–1, 1–3, 3–10, and >10 Mb fragments were 0, 13.9, 32.0, and 82.6%, respectively, and the positive detection rates in samples with 0–1, 1–3, 3–10, and >10 Mb fragments were 0, 11.1, 34.8, and 85.0%, respectively (Figure 6).

## Discussion

China is a country with a high incidence of birth defects. At present, the incidence of birth defects is approximately 5.60%, and the number of new birth defects is approximately 900,000 every year. Birth defects, resulting from chromosomal aneuploidies and CNVs, have gradually become the main cause of infant death and child disability (Zhang and Zhang, 2013). However, NIPT has not been widely used in fetal CNV detection. One of the main reasons is the high cost of deep sequencing. Currently, the read depth of the BGISEQ-500 sequencing platform has been upgraded from 6 to 25 M, while the price is basically unchanged. This introduces a great opportunity to extend fetal CNV detection to a large scale.

Amniocentesis is a key restrictive step in the diagnosis of birth defects. However, reliable genetic counseling on test selection for prenatal screening and diagnosis is difficult because of the possible risk of infection and subsequent abortion by amniocentesis. Patients often suffer financial expenses and unnecessary and excessive concern, which contribute to a consensus that the psychological trauma of pregnant women has a negative impact on fetal development (Finlay-Jones et al., 2015; Verbeek et al., 2015; Bell et al., 2016; Burden et al., 2016). The low positive fetal CNV detection rate in NIPT can lead to a large number of unnecessary amniocentesis tests, which might cause a shortage of medical resources and restrict reductions in birth defects. Thus, increasing the positive detection rate in NIPT and reducing the frequency of amniocentesis will greatly contribute to reducing the risk of birth defects.

In this study, an increase in the read depth from 4–6 to 25 M correlated with a reduction (from 21 to 5) in the number of fetuses identified with suspected CNVs. In other words, increased read depth helped eliminate 76.19% of false positive cases and increase the diagnostic efficiency by 4.20 times.

Furthermore, we also compared the CNV detection rates of two different sequencing platforms under low coverage. BGISEQ-500 is a high-throughput sequencing solution, powered by combinatorial Probe-Anchor Synthesis (cPAS) and improved DNB^TM^ technology. cPAS chemistry works by incorporating a fluorescent probe into a DNA anchor on the DNB, followed by high-resolution digital imaging. This combination of linear amplification and DNB technology reduces the error rate while enhancing the signal. In addition, the size of the DNB is controlled in such a way that only one DNB is bound per active site. This patterned array technology not only provides sequencing accuracy but also increases chip utilization and sample density.

DA8600 is another high-throughput sequencing solution, powered by a semiconductor sequencing system and proton-pH sensing technology. DNA strands are fixed in the micropores of semiconductor chips by the semiconductor sequencing system. Then, DNA polymerase synthesizes complementary DNA strands from the fixed DNA strands. Each extension of the DNA strand releases a proton, which causes a local pH change. Ion sensors detect these pH changes and convert chemical signals into digital signals, which allow the real-time identification of bases. Hence, both BGISEQ-500 and DA8600 represent industry-leading high-throughput sequencing platforms that are able to detect common chromosomal aneuploidies such as T13, T18, and T21 with low false positive and false negative rates. However, in this study, the BGISEQ-500 sequencing platform had a 3.78 times higher positive screening rate (20/4721, 0.42%) than the DA8600 sequencing platform (8/7182, 0.11%) for fetal CNV detection. Meanwhile, the positive CNV detection rates in NIPT by the BGISEQ-500 and DA8600 sequencing platforms were almost the same (13.33 and 16.67%, respectively). The above results show that the BGISEQ-500 sequencing platform might be more suitable for fetal CNV detection in NIPT.

Pathogenic CNVs are small and can be undetectable with conventional karyotyping. Wang et al. (2017) calculated 112 submicroscopic pathogenic CNVs detected among fetuses with abnormal ultrasound findings; of these, 29 were less than 1 Mb in size, 66 were between 1 and 5 Mb, 11 were 5–10 Mb, and only six were larger than 10 Mb. The mean sizes of microdeletions and microduplications were 2.9 ± 2.6 Mb and 3.0 ± 3.7 Mb (mean ± SD), respectively. This suggests that NGS at a read depth of 25 M is more suitable for fetal CNV detection than conventional karyotyping; therefore, the combination of NGS with a high read depth and amniocentesis with subsequent CMA is a useful tool for fetal CNV detection to reduce birth defects. In the future, NGS with a high read depth may replace previous detection methods in NIPT.

In this study, we also found that CNVs (1–3, 3–10, and >10 Mb) could be effectively detected by NGS, suggesting that both NGS and CMA are suitable for CNV detection. In addition, although karyotype analysis adequately detected CNVs in large fragments (>10 Mb), it had obvious disadvantages in the detection in small fragments (0–1, 1–3, and 3–10 Mb). However, with increased read depth and reduced sequencing cost, NGS has more advantages for CNV detection.

## Conclusion

In summary, NIPT by NGS is a potential method for fetal CNV detection, although this technique requires further investigation. Increasing read depth is essential to help reduce the false positive CNV detection rate and frequency of unnecessary amniocentesis for suspected CNVs.

## Materials and Methods

### Subjects for Comparative Analysis of 4–6 M Reads and 25 M Reads in NIPT

Within a 15-month period (March 2017 to June 2018), 11,903 pregnant women were enrolled at the International Peace Maternity and Child Health Hospital (IPMCH) to undergo NIPT by NGS at read depths of 4–6 M (Supplementary Table 1). The study was conducted with the approval of the Hospital Ethics Committee of the IPMCH. Fetuses with suspected CNVs were further evaluated by amniocentesis and CMA. Subsequently, the samples were re-sequenced at a read depth of 25 M.

Inclusion criteria for NIPT were as follows: high-risk values (T21 ≥ 1/270, T18 ≥ 1/350) or critical-risk values (1/1,000 ≤ T21 < 1/270, 1/1,000 ≤ T18 < 1/350) at maternal serological screening, advanced maternal age (35 years or older at the expected date of delivery), abnormal fetal structure identified by ultrasound, adverse pregnancy history, or partial molar pregnancy involving anxiety and where the mother volunteered for NIPT.

The exclusion criteria were in accordance with the rules of the Chinese government and were as follows: twin or higher-order pregnancy, chromosomal abnormality in either parent of the fetus, a history of embryo transplantation or stem cell therapy, and immunotherapy within 1 month or allogeneic blood transfusion within 1 year.

### Subjects for Comparative Analyses by NGS at 25 M Reads and CMA

Within a 27-month period (November 2016 to January 2019), 6,763 pregnant women were enrolled at the IPMCH to undergo screening for fetal chromosomal abnormalities by CMA, and fetus samples with identified CNVs were re-sequenced at 25 M reads by NGS (Supplementary Table 2). We then analyzed the positive CNV detection rates in CMA and NGS.

### Collection and Treatment of Blood Samples

Maternal peripheral blood samples (5 mL) were collected in EDTA tubes, fully mixed, and refrigerated temporarily at 4°C. Samples were excluded if they showed hemolysis or were stored more than 8 h before plasma separation. Each blood sample was centrifuged at 1,600 × *g* for 10 min at 4°C, and the plasma was carefully collected and dispensed into 2.0-mL Eppendorf tubes. The plasma was centrifuged again at 16,000 × *g* for another 10 min at 4°C. The upper plasma was carefully divided into clean 2.0 mL Eppendorf tubes, each containing approximately 600 mL of plasma, and refrigerated at −80°C. Repeated freezing and thawing of the plasma before experiments was minimized.

### NIPT on the BGISEQ-500 Sequencing Platform

DNA extraction, library construction, and sequencing were performed according to the standard protocols of the Human Molecular Genetics Guidelines of the IPMCH. Maternal plasma (200 mL) was used for cffDNA extraction using BGISP-300 (BGI, Shenzhen, China) and a Nucleic Acid Extraction Kit (BGI). After DNA extraction, end repair was conducted by adding end-repair enzymes with the following cycle conditions: 37°C for 10 min and 65°C for 15 min, followed by adaptor ligation at 23°C for 20 min with label-adaptor and ligase. After end-repair and adaptor ligation, PCR was used to amplify DNA to the desired concentration with the following cycle conditions: 98°C for 2 min, then 12 cycles at 98°C for 15 s, 56°C for 15 s, and 72°C for 30 s, with a final extension at 72°C for 5 min.

The DNA amplification products were quantified by Qubit^®^ 2.0 (Life Technologies, Invitrogen, Rockville, MD, United States) using Qubit^TM^ dsDNA HS Assay Kits (Life Technologies), and concentrations of 2 ng/μL or more were regarded as qualified standards. The volume was calculated according to the concentration of each sample, and samples of the same mass were pooled. The DNA double strands were thermally denatured into single strands after pooling, and then cyclic buffer and ligase were added to generate DNA circles *via* cyclization. The DNA circles were used to create DNA Nanoballs^TM^ (DNBs) by rolling circle replication. The concentration of DNBs was quantified by Qubit^®^ 2.0 using Qubit^®^ ssDNA Assay Kits (Life Technologies), and DNB concentrations in the range of 8–40 ng/μL were considered as appropriate. The DNBs were loaded onto chips and sequenced on the BGISEQ-500 sequencing platform (BGI). Any sample that failed to meet the quality control criteria was reported as a detection failure by NIPT.

Algorithms developed by BGI-Shenzhen were used to detect CNV. In brief, the reads were aligned to the human genome (hg19) using SOAP2. The non-unique mapped and PCR duplication reads were removed. The genome was divided into 100-kb non-overlapping windows, and the reads number was counted separately as the original effective data of each window. After GC correction, intra-batch correction and Principal Component Analysis (PCA), hidden states of each window (normal, deletion and duplication) were inferred using Hidden Markov Model (HMM), and then the CNV is finally identified.

## Data Availability Statement

The original contributions presented in the study are included in the article/Supplementary Material, further inquiries can be directed to the corresponding author/s.

## Ethics Statement

The studies involving human participants were reviewed and approved by the Hospital Ethics Committee of the International Peace Maternity Child Health Hospital of China Welfare Institute (IPMCH). The patients/participants provided their written informed consent to participate in this study.

## Author Contributions

DL, CX, and SC conceived and designed the experiments. DL, CX, SC, and LZ performed the experiments. SC, SL, CC, and YC collected the data. HF, JZ, YW, and HH contributed to new materials. JG and SC analyzed the data and wrote the manuscript. All authors read and approved the manuscript.

## Conflict of Interest

The authors declare that the research was conducted in the absence of any commercial or financial relationships that could be construed as a potential conflict of interest.

## Supplementary Material

Supplementary Table 1 Patient data for NIPT.

Supplementary Table 2 Patient data for CMA.
